# Supplementary material for: Human RIG-I Antiviral Deficiency Caused by a Dominant-Negative Variant Locked in a Signaling-Inactive State
Source: medRxiv. 2026 Mar 6:2026.03.02.26347088. Preprint. [Version 1] doi: 10.64898/2026.03.02.26347088 (PMC12976907; doi:10.64898/2026.03.02.26347088)
Supplement: Supplement 1 [file media-1.pdf]

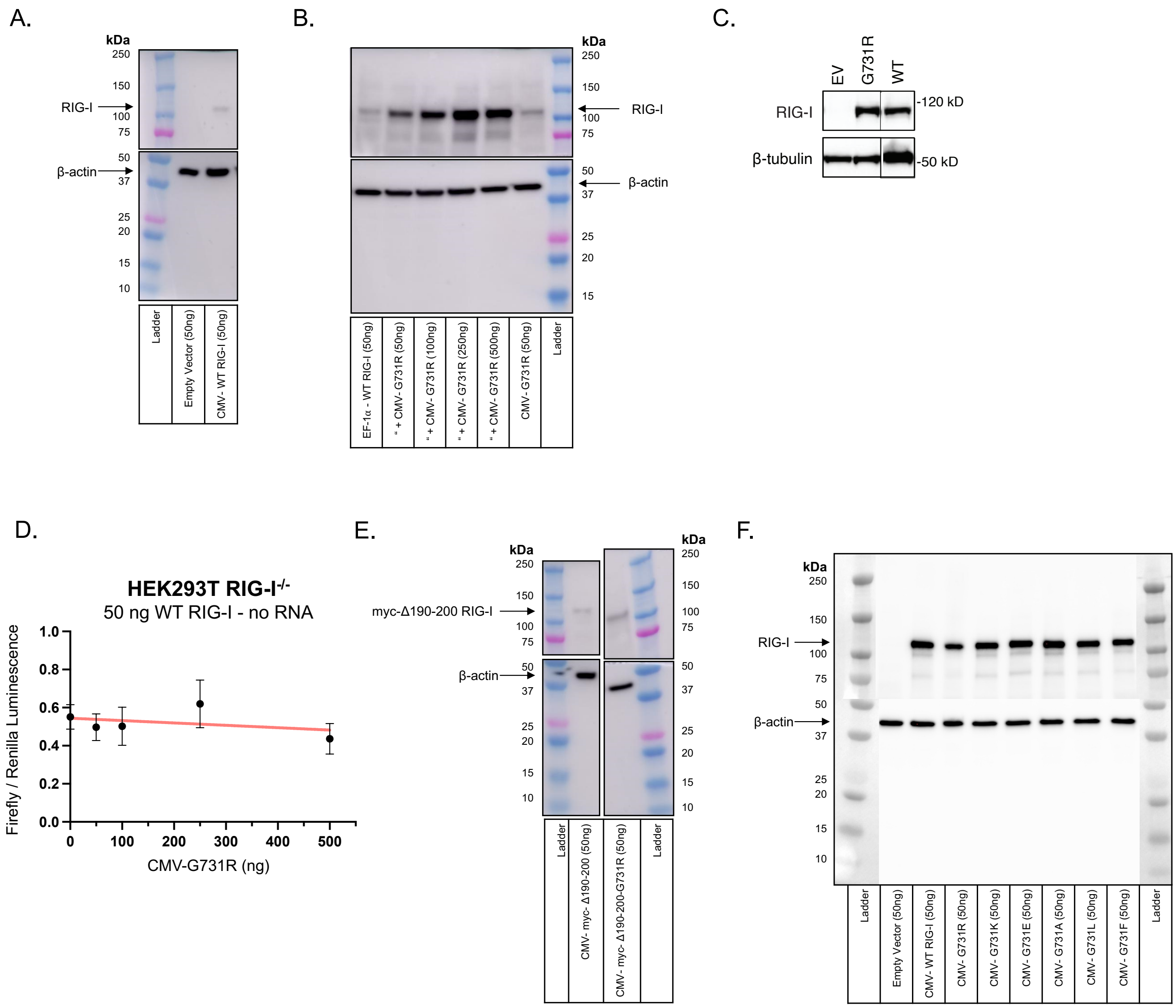

Figure S1. IFN-β reporter assay western blots and control

Western blot corresponding to IFN-β reporter assay in HEK293T RIG-I$^{-/-}$ cells for transient expression of (A) CMV- WT RIG-I, (B) EF1-α- WT RIG-I and competing CMV-G731R, (E) CMV- myc-Δ190-200 constructs, and (F) CMV- G731X mutants relative to β-actin. Panels (A) and (B) are related to data in figure 2, (E) is related to figure 6, and (F) is related to figure 7.

(C) Immunoblot assessing expression of RIG-I WT and G731R, corresponding to Figure 2B. Cell lysates from transfections with empty vector (EV), RIG-I WT, or RIG-I G731R were resolved on the same gel. A section of the gel between the WT and G731R lanes was removed, as indicated by the black line.

(D) IFN-β response of constitutively expressed WT RIG-I under EF1-α promoter measured by dual luciferase reporter assay in the absence of RNA, while titrating G731R plasmid, related to data in figure 5. Each condition was performed three times (technical replicates, n=3). The plot shown represents the mean values at each protein concentration with error bars representing the standard error of the mean (SEM), and the curve represents hyperbolic decay fitting of the mean data.

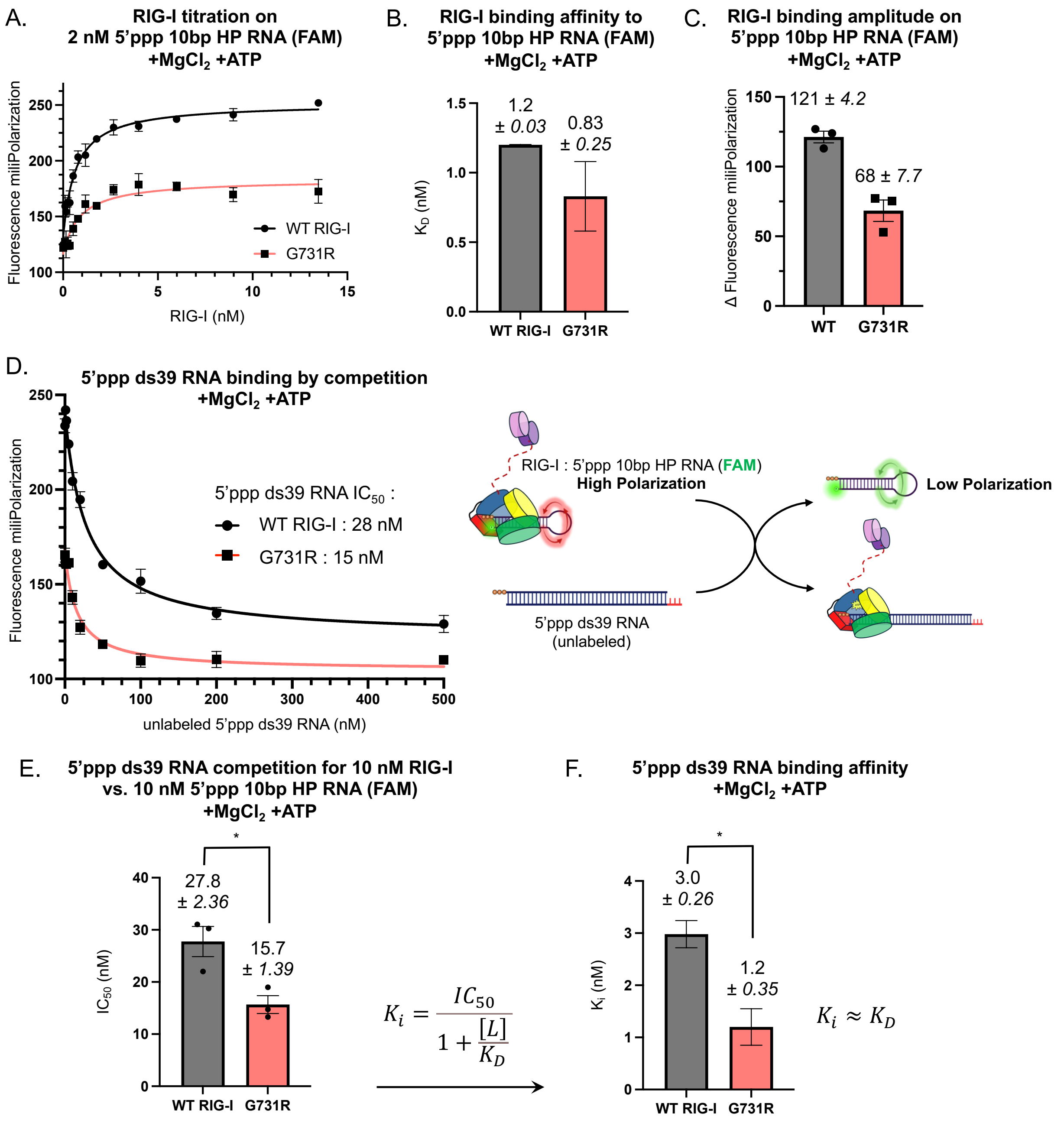

Figure S2. Fluorescence-based RNA binding measurements related to RNA binding data in figure 3B.

(A) Fluorescence polarization (FmP) of fluorescein-labeled 5'ppp 10bp HP RNA in the presence of increasing protein concentration (technical replicates, n=3). The RNA binding data were fit to equation 2 to obtain the FmP amplitude and the RNA K_D values .

(B) 5'ppp 10bp HP RNA binding affinity (K_D) measured by changes in fluorescence polarization while titrating RIG-I against constant 2 nM RNA. Error bars represent the standard error of the mean (technical replicates, n=3).

(C) The FmP amplitude values from experiments in panel A are compared (technical replicates, n=3).

(D) 5'ppp ds39 RNA binding to WT or G731R was measured by competition assay. Fluorescence polarization of FAM-labeled 5'ppp 10bp HP RNA (10 nM) bound to RIG-I or G731R (10 nM) was measured while titrating in increasing unlabeled 5'ppp ds39 RNA. The data were fit to equation 1 to obtain the half maximal inhibitory concentration IC_{50}. Error bars represent the standard error of the mean (technical replicates, n=3).
(E) 5'ppp ds39 RNA competition (IC_{50}) for RIG-I displacement from 5'ppp 10bp HP RNA as monitored by changes in fluorescence polarization while titrating 5'ppp ds39 RNA against 10 nM equimolar RIG-I-bound 5'ppp 10bp HP RNA (FAM). Error bars represent the standard error of the mean (technical replicates, n=3),

(F) Cheng-Prusoff equation is used to obtain 5'ppp ds39 RNA K_i values, which are representative of RIG-I's binding affinity for 5'ppp ds39 RNA (K_D). Error bars represent the propagated standard error of the means. Statistical analysis was performed using unpaired t-test. *, $P<0.05$.

Consolidated HDX differential peptide coverage

Δ HDX							
**Domain**	**Feature**	**Peptide Sequence**	Start Residue	End Residue	wt RIG-I ± 5'ppp 10bp HP RNA	apo G731R vs. WT RIG-I	G731R ± 5'ppp 10bp HP RNA
CARD1 (1-91)		FQDYIRKTLDPTYIL	11	25	15 (4)	2 (3)*	-17 (2)
		FQDYIRKTLDPTYILS	11	26	15 (4)	-1 (2)*	-21 (3)
		YIRKTLDPTYIL	14	25	15 (5)	2 (2)*	-20 (3)
		SYMAPWFREEEVQ	26	38	17 (4)	1 (2)*	-30 (3)
		YIQAEKNNKGPMEAATL	39	55	11 (4)	-6 (3)	-28 (3)
		LKFLLE	57	62		0 (1)*	2 (2)*
		QEEGWFRGFLDA	64	75	13 (5)	-8 (2)	-17 (3)
		DALDHAGYSGL	74	84	8 (2)	-6 (3)	-22 (4)
		DALDHAGYSGL	74	84	12 (3)	-6 (3)	-21 (4)
		YEAIESWD	85	92	16 (5)	2 (3)*	-28 (4)
CARD2 (92-185)		FKKIEKLEE	93	101	-4 (7)*	-5 (3)	-40 (3)
	CARD2:Hel2i latch (100-115)	YRLLL	102	106	11 (3)	6 (3)	
		KRLQPEF	107	113	6 (4)	6 (2)	-27 (3)
		KTRIIPTDIISDL	114	126	17 (4)	1 (3)*	-33 (3)
		KTRIIPTDIISDL	114	126	17 (3)	-2 (3)*	-38 (2)
		LINQECEE	130	137		-2 (3)*	-31 (5)
	CARD2:Hel2i latch (145-150)	ILQICSTKGMMAGAEKLVE	138	156	22 (3)	2 (2)*	-19 (4)
		ILQICSTKGMMAGAEKLVE	138	156	16 (4)	2 (1)*	-14 (3)
		LRSDKENWPKTLKLAL	159	174	22 (8)	3 (2)*	-30 (2)
		LRSDKENWPKTLKLAL	159	174		3 (2)*	-29 (4)
		ALEKERNKFSEL	173	184	6 (4)	1 (3)*	-37 (5)
ID Linker (185-244)		WIVEKGIKDVETEDL	185	199	2 (4)*	-8 (2)	-57 (5)
	Electrostatic gate (190-220)	WIVEKGIKDVETEDLEDKMETSD	185	207	1 (2)*		-46 (6)
		EDKMETSDIQIF	200	211	4 (6)*		-25 (3)
		YQEDPECQNL	212	221	3 (4)*	-8 (3)	-54 (7)
		SENSCPPSEVSDTNL	222	236	4 (4)*	-7 (2)	-80 (4)
		VSDTNL	231	236	2 (3)*	-15 (3)	-88 (5)
		YSPFKPRN	237	244	0 (9)*	-12 (2)	-88 (4)
Hel1 (244-455)		YSPFKPRNYQLE	237	248	0 (6)*		-55 (4)
		LALPAMKGKNTIICAPTGCGKTFVSL	249	274	-1 (3)*	-3 (2)*	-33 (2)
		TIICAPTGCGKTF	259	271	2 (5)*	-5 (1)*	-56 (4)
	RNA binding motifs:	LICEHHLKKFPQGQKGKVVF	275	294		-1 (1)*	-23 (2)
	motif Ia (300-330)	FANQIPVYE	295	303	-16 (4)	-11 (3)	
		FERHGYRVTGISGATAENVPVEQ	313	335	-3 (6)*	-4 (2)*	-54 (4)
		FERHGYRVTGISGATAENVPVEQ	313	335	-2 (7)*	-5 (1)*	-37 (2)
		IVENND	336	341	-2 (2)*	1 (2)*	-7 (3)
	motif Ic (340-370)	IIILTPQIL	342	350	-9 (4)	3 (2)*	-7 (3)
		IIILTPQILVNNLKKGTIPSL	342	362	-12 (5)	-1 (2)*	-36 (3)
		VNNLKKGTIPSLSIF	351	365	-7 (4)	-2 (2)*	-36 (3)
	motif IIa (370-390)	MIFDECHNTSKQHPYNM	368	384	-9 (4)	-1 (2)*	-22 (2)
		IFDECHNTSKQHPYNMIM	369	386	-7 (3)	2 (1)*	-20 (2)
		DECHNTSKQHPYNMIM	371	386	-9 (4)	0 (1)*	-27 (3)
		FNYLDQKLGGSSGPLPQVIGL	387	407	0 (2)*	2 (1)*	-22 (2)
		FNYLDQKLGGSSGPLPQVIGL	387	407	0 (2)*	2 (1)*	-20 (2)
		TASVGVGDAKNTDEAL	408	423	-4 (3)*	-2 (3)*	
		DYICKL	424	429		3 (2)*	2 (5)*
		DASVIATVKHNLEEL	434	448	0 (3)*	0 (3)*	-8 (2)
		SVIATVKHNLEEL	436	448	-1 (4)*	1 (3)*	-12 (2)
Hel2 (245-469)...	Insertion domain linker	LEQVVYKPQKF	448	458	-4 (5)*	-2 (3)*	-41 (5)
		LEQVVYKPQKF	448	458	-3 (5)*	1 (3)*	-36 (4)
		EQVVYKPQKF	449	458	-4 (5)*	0 (3)*	-28 (3)
Hel2i (470-607)		FRKVESRISDKFKYIIAQL	459	477		-1 (2)*	-20 (2)
		MRDTESLAKRICKDLENL	478	495		-2 (2)*	-13 (3)
		AKRICKDLENL	485	495	-7 (5)	-2 (3)*	-21 (5)
	RNA binding region (505-520)	SQIQNREFGTQKYEQW	496	511	-4 (4)*	0 (2)*	-43 (4)
		IVTVQKACM	512	520	-2 (2)*	-7 (2)	-3 (4)*
		IVTVQKACM	512	520	-1 (5)*		-1 (2)*
	CARD2/CTD :Hel2i latch (520-540)	VFQMPDKDEESRICKA	521	536	-12 (5)	-4 (3)*	-31 (3)
		VFQMPDKDEESRICKA	521	536	-10 (4)	-1 (3)*	-29 (3)
		VFQMPDKDEESRICKALF	521	538	-8 (4)	-2 (2)*	-22 (3)
		LYTSHLRKYNDAL	539	551	0 (1)*	2 (1)*	2 (2)*
		DALIISEHARMKD	549	561	1 (3)*	0 (1)*	-6 (2)
	CARD2:Hel2i latch (565-580)	HARMKDALD	556	564	8 (50)	-1 (1)*	-12 (3)
		HARMKDALD	556	564	8 (49)	-1 (1)*	-14 (4)
		YLKDFFSNVRAAGF	565	578	10 (3)	-1 (2)*	-25 (3)
		ESVSRDPSNENPKLED	597	612	1 (4)*	-3 (2)*	-28 (3)

Consolidated HDX differential peptide coverage (cont.)

Domain	Feature	Peptide Sequence	Start Residue	End Residue	Δ HDX: wt RIG-I ± 5'ppp 10bp HP RNA	Δ HDX: apo G731R vs. WT RIG-I	Δ HDX: G731R ± 5'ppp 10bp HP RNA
Hel2 (608-743)	RNA binding motifs	VSRDPSNENPKLEDLCF	599	615	-2 (3)*	-1 (3)*	-24 (2)
		FILQEEYHLNPET	615	627	0 (4)*	-2 (2)*	-10 (2)
		ILQEEYHLNPET	616	627	-1 (2)*	-1 (2)*	-13 (2)
	motif IV (630-695)	VKTRALVDALKN	633	644	-7 (2)	-3 (1)*	-15 (3)
		ALKNWIEGNPKL	641	652	1 (3)*	-2 (2)*	-13 (2)
		ALKNWIEGNPKL	641	652	1 (3)*	-1 (2)*	-12 (2)
		SFLKPGIL	653	660	-8 (2)	1 (2)*	-18 (3)
		TGRGKTNQNTGMTLPAQKCIL	661	681	-9 (4)	-11 (2)	-81 (3)
		DAFKASGDHNIL	682	693	-9 (3)	0 (3)*	-53 (4)
		DAFKASGDHNIL	682	693	-9 (3)	-5 (2)*	-42 (3)
		FKASGDHNIL	684	693	-6 (3)	0 (2)*	-35 (3)
		FKASGDHNIL	684	693	-6 (3)	0 (2)*	-34 (2)
	motif V (695-720)	IATSVADEGID	694	704	-14 (3)	-5 (2)*	-41 (5)
		IATSVADEGID	694	704	-13 (4)	-4 (2)*	-35 (3)
		IAQCNL	705	710	-7 (4)	-3 (3)*	-40 (3)
		VILYE	711	715	-1 (1)*	1 (2)*	-2 (3)*
		YEYVGNVIKM	714	723	-14 (4)	-4 (3)*	-38 (3)
		FLLTSNA	738	744	-9 (5)	-6 (1)	-33 (3)
Pincer (744-803)	Helicase bridge / CTD linker	LTSNAGVIEKEQINM	740	754	-3 (4)*	-8 (3)	-19 (4)
		YKEKMMNDSIL	755	765	0 (1)*	5 (2)*	-7 (2)
		QTWDEAVF	768	775	-1 (3)*	-1 (2)*	-41 (4)
		WDEAVF	770	775	1 (2)*	-1 (3)*	-18 (2)
CTD (804-925)		VRVIEECHY	822	830	-2 (4)*	-2 (1)*	-14 (2)
		TVLGDA	831	836	0 (3)*		-30 (3)
	5' capping loop (840-860)	TVLGDAFKECF	831	841	-2 (4)*	0 (2)*	
		VSRPHPKPKQFSSF	842	855		-6 (1)	-80 (3)
		CARQNCSHDWGIHVKYKTFEIPVIKIESF	863	891		-1 (1)*	-9 (3)
	RNA binding region (880-905)	VVEDIATGVQTL	892	903	-11 (3)	-6 (3)	-30 (3)
		DIATGVQTL	895	903	-10 (3)	-8 (3)	-28 (6)
		YSKWKDFHF	904	912		-6 (2)	-33 (3)
		EKIPFDPAEMSK	913	924	-2 (3)*	-7 (2)	-38 (5)
		EKIPFDPAEMSK	913	924	-1 (4)*	-7 (2)	-53 (3)

Figure S3. Annotated mapping of consolidated HDX differential peptide coverage related to data in figure 4.

Chart summarizing the differential HDX patterns of specific peptide of RIG-I from each experimental condition, annotated by protein domain and functional features.

Figures S4-S9. Hydrogen-Deuterium Exchange Mass Spectroscopy sequence coverage heatmaps related to data in figure 4.

Time-consolidated HDX data is shown using a perturbation view from HDX Workbench (Scripps Research Institute, 2024). Individual peptides are represented by horizontal strips below their respective portion of the protein sequence with values representing the percent deuterium uptake ± standard deviation. Individual peptides are colored according to the colorimetric perturbation key shown.

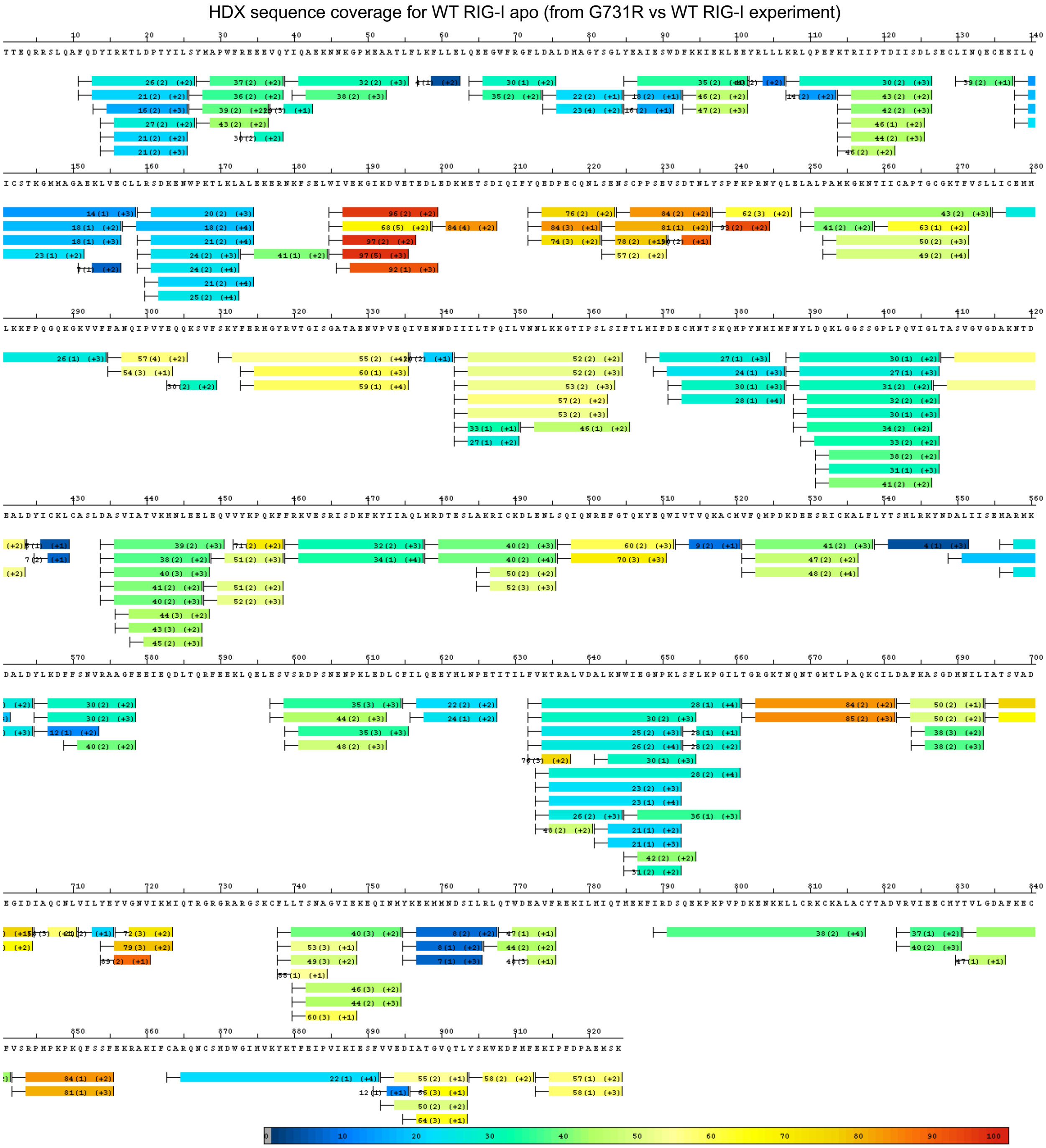

Figure S4. HDX sequence coverage for WT RIG-I apo (from G731R vs WT RIG-I experiment)

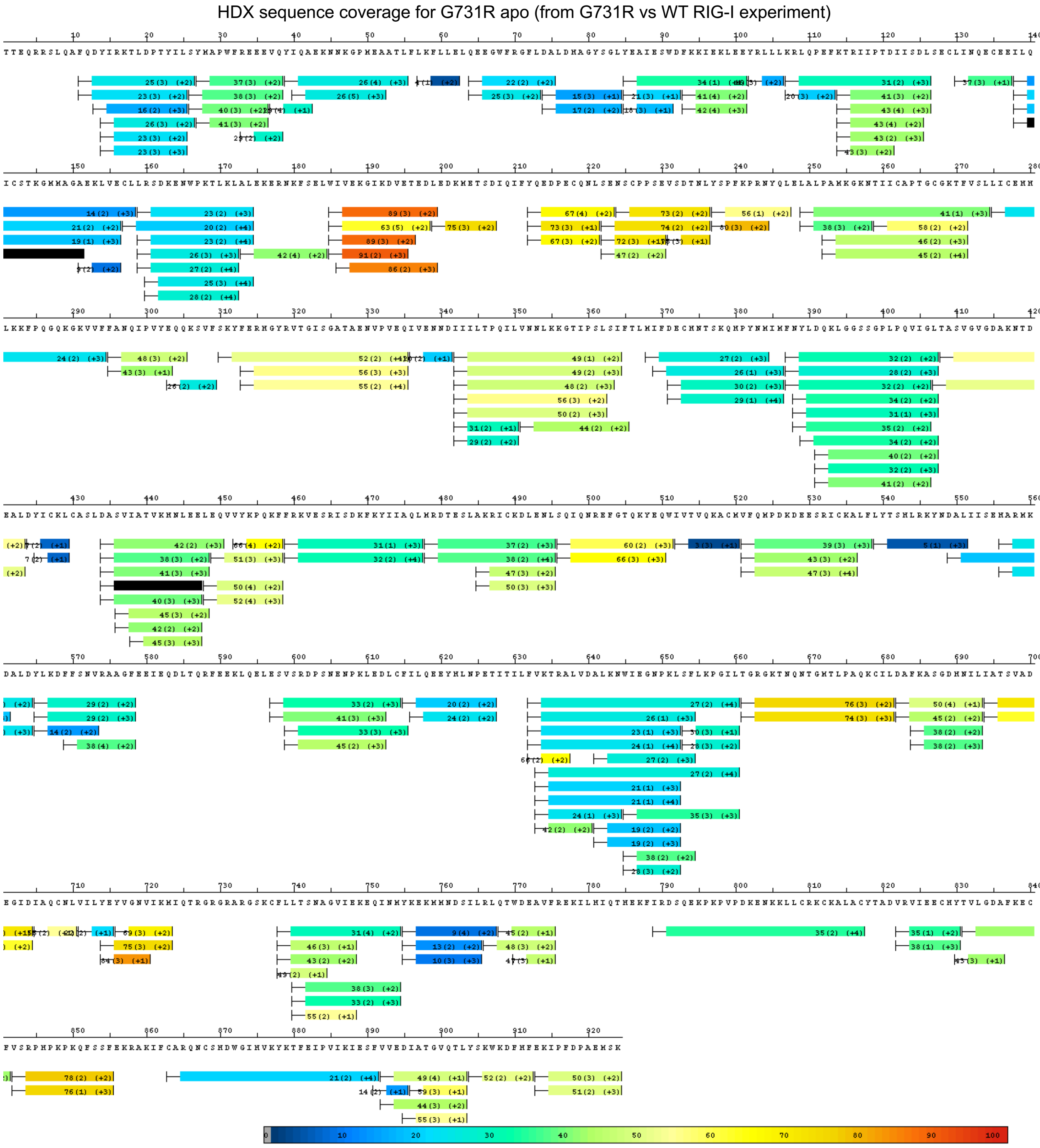

Figure S5. HDX sequence coverage for G731R apo (from G731R vs WT RIG-I experiment)

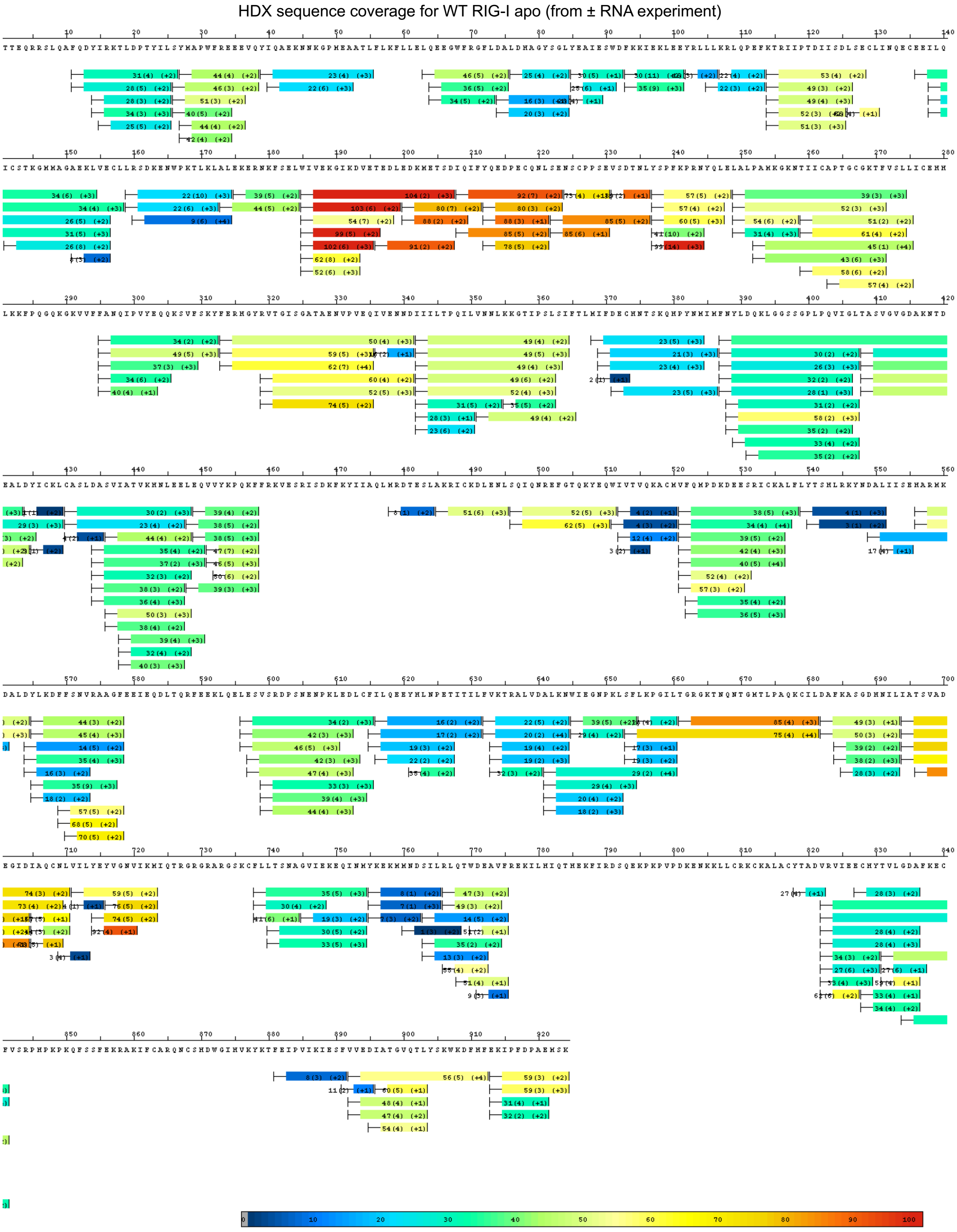

Figure S6. HDX sequence coverage for WT RIG-I apo (from ± RNA experiment)

HDX sequence coverage for WT RIG-I + RNA (from ± RNA experiment)

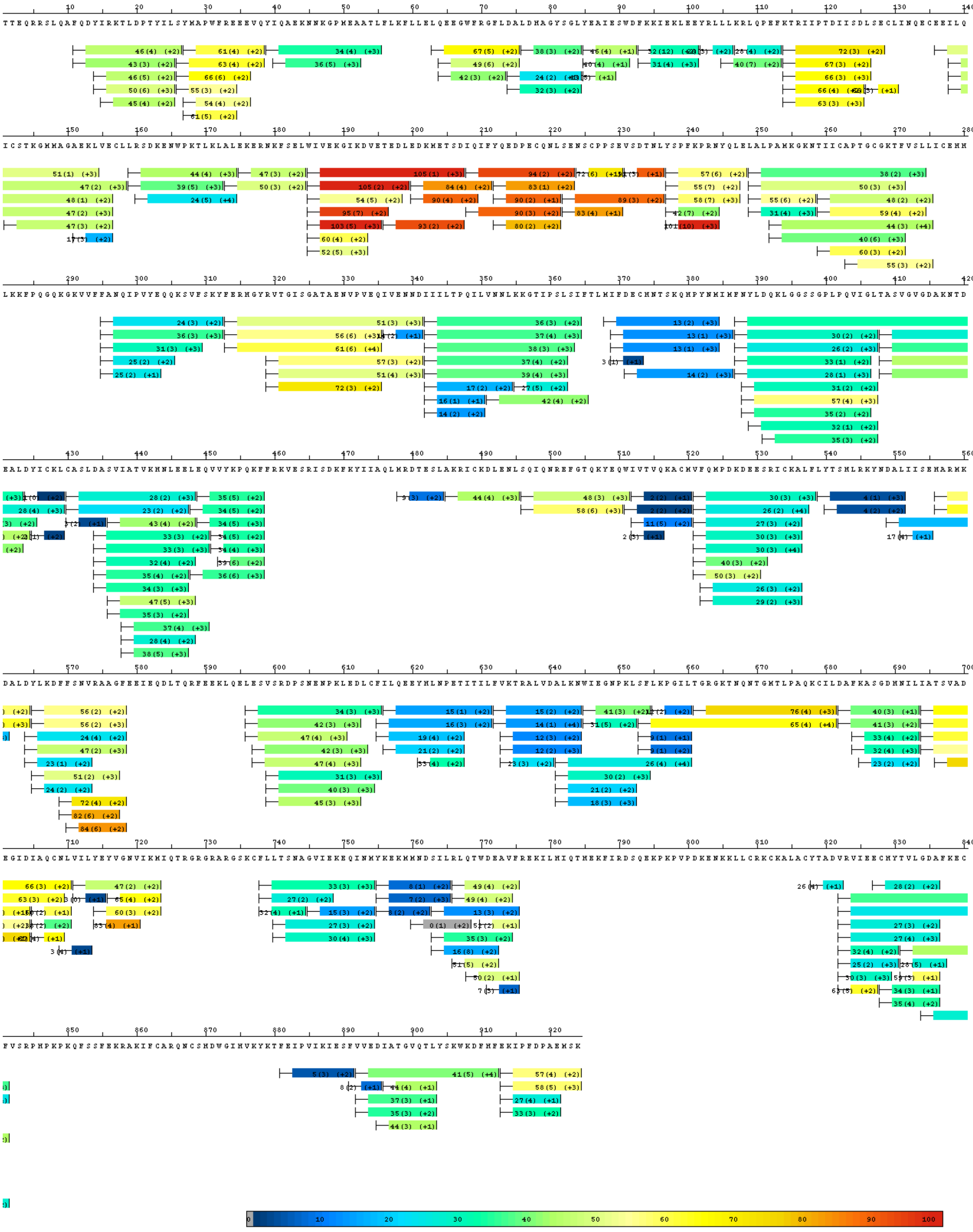

Figure S7. HDX sequence coverage for WT RIG-I + RNA (from ± RNA experiment)

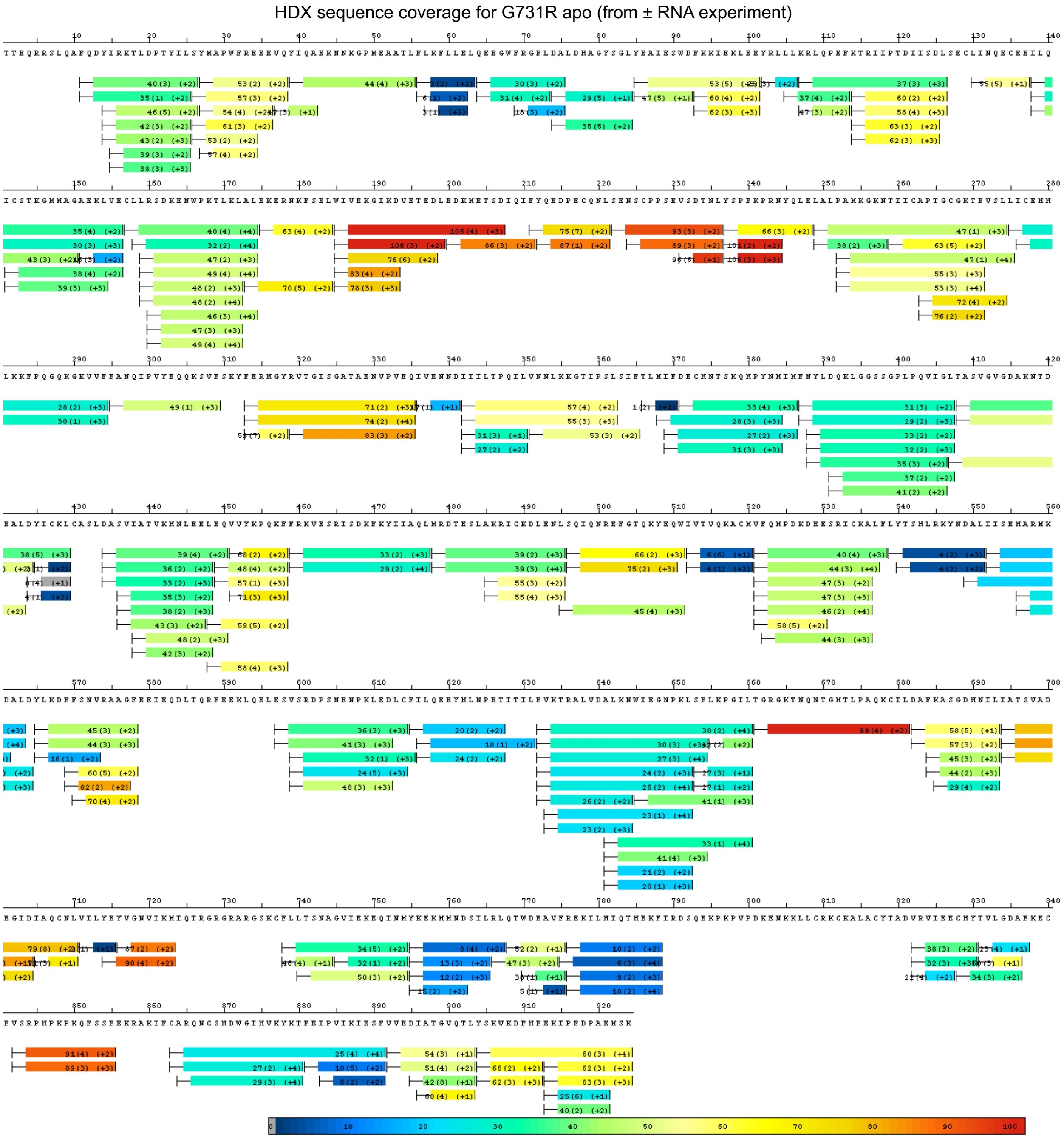

Figure S8. HDX sequence coverage for G731R apo (from ± RNA experiment)

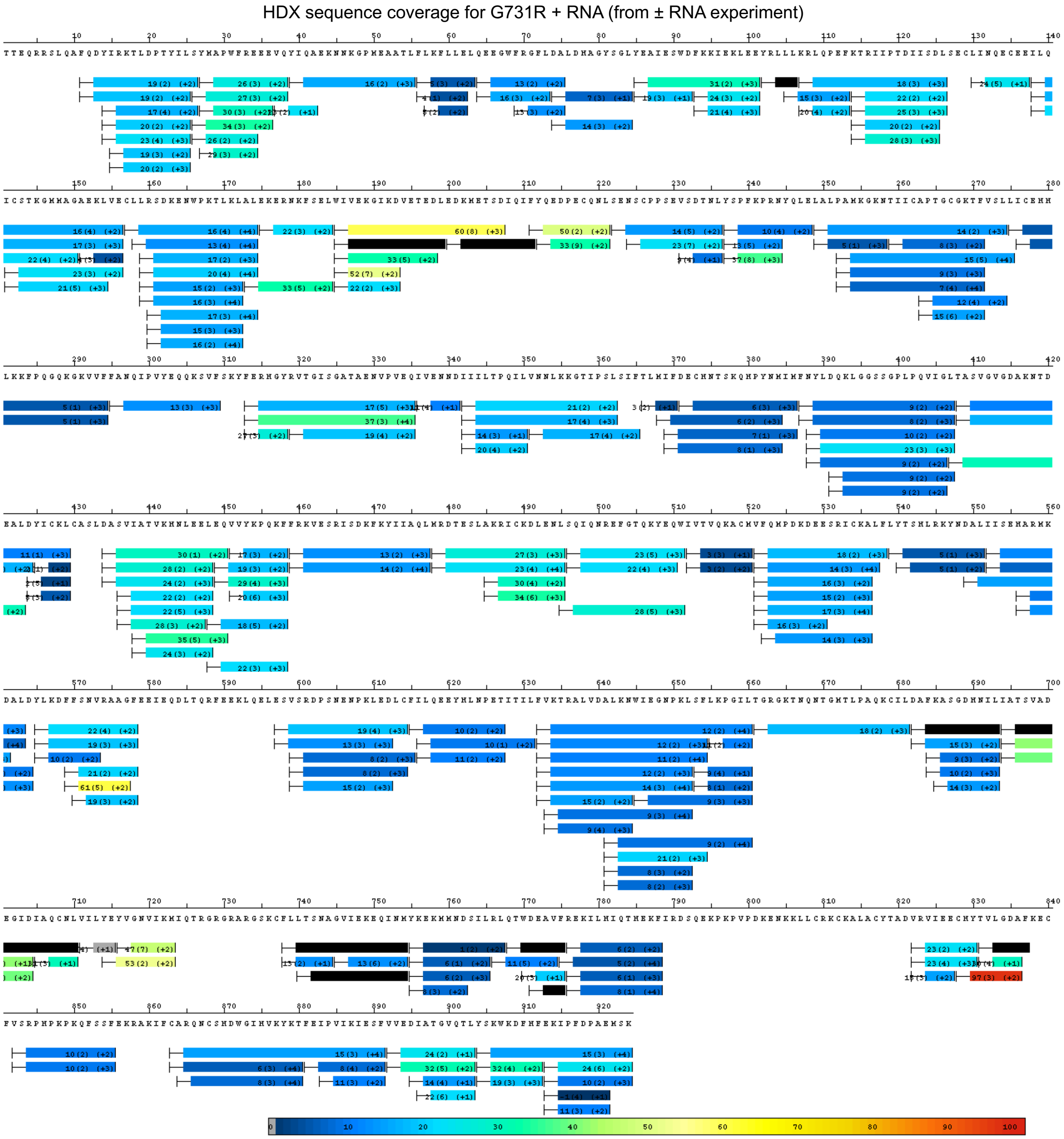

Figure S9. HDX sequence coverage for G731R + RNA (from ± RNA experiment)

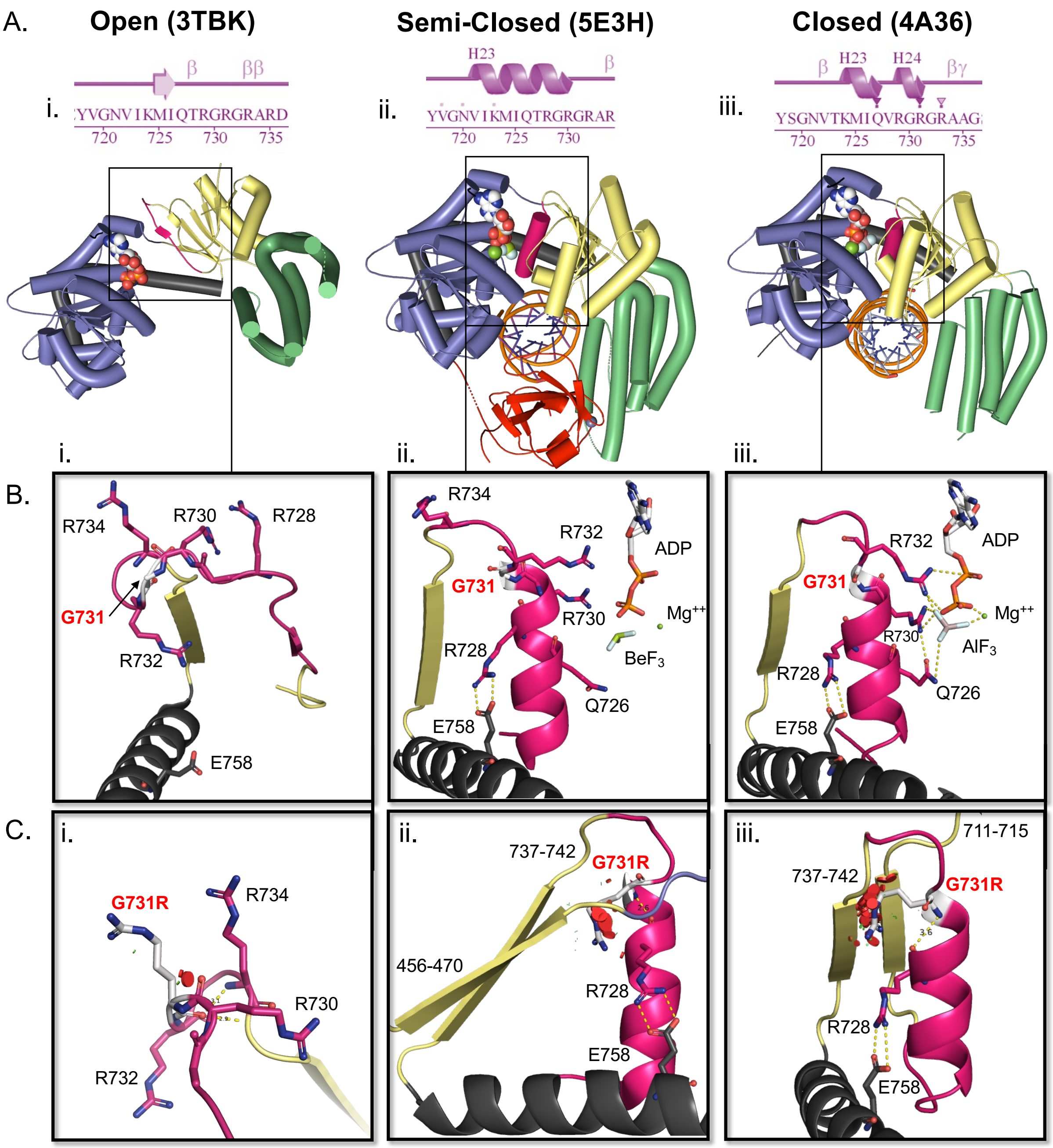

Figure S10. Conformational changes in RIG-I from open to closed states upon RNA binding

(A) PDBsum linear representation of secondary structure formation in motif VI (in pink) of (i) open mouse RIG-I helicases (PDB: 3TBK), (ii) semi-closed human RIG-I (PDB: 5E3H), and (iii) closed duck RIG-I helicases (PDB: 4A36).Below are front top views of

RIG-I crystal structures captured in each conformational state. Structures are aligned by the Hel1 domain.

(B) Zoomed in view of each respective RIG-I structure showing the isolated motif VI and associated interactions in detail.

(C) PyMOL Mutagenesis Wizard analysis of G731R computational mutation in each respective structure. The red disks indicate pairwise overlap of atomic van der Waals radii.

Table S1: Whole Genome Sequencing analysis summary
(see excel file)

Table S2: RNA binding measurements of WT RIG-I and G731R by fluorescence polarization were determined from experiments shown in figures 3B and S2.

RNA	constant	WT RIG-I	G731R
5'ppp 10bp HP (FAM)	K_D	1.2 ± 0.03 nM	0.83 ± 0.25 nM
5'ppp ds39	IC_{50}	27.8 ± 2.4 nM	15.7 ± 1.4 nM
5'ppp ds39	K_i	3.0 ± 0.3 nM	1.2 ± 0.4 nM

Table S3: RNA unbinding measurements of WT RIG-I and G731R by stopped-flow fluorescence intensity were determined from experiments shown in figure 5 C,D,E.

	WT		G731R		
	CTD	Helicase	CTD	Helicase	
k_{off} (s^{-1})	0.141 ± 0.011	0.0045 ± 0.00006	0.147 ±0.015	0.0062 ± 0.003	**- ATP**
Kinetic mode population (%)	75.1 ± 4.26	24.9 ± 4.26	92.4 ± 2.03	7.6 ± 2.03	
Kinetic mode lifetime (s)	7.1 ± 0.5	223 ± 2.8	6.7 ± 0.7	198 ± 76.0	
R^2	0.9829 ± 0.01123		0.9814 ± 0.01063		
k_{off} (s^{-1})	0.128 ± 0.012	0.0105 ± 0.001	0.137 ± 0.010	0.0096 ± 0.006	**+ ATP**
Kinetic mode population (%)	76.7 ± 0.99	23.3 ± 0.99	95.2 ± 0.74	4.8 ± 0.74	
Kinetic mode lifetime (s)	7.9 ± 0.7	96 ± 10.9	7.4 ± 0.5	176 ± 127	
R^2	0.9875 ± 0.007793		0.9822 ± 0.007674		

Table S4: Comparison of CADD and AlphaMissense scores to biochemical impact, as determined from experiments shown in figure 7A, for selected G731 missense mutants.

Genomic DNA change	cDNA change	Protein variant	Original	Nucleotide changed	CADD score	AlphaMissense score	AlphaMissense class	MAF in gnomAD	IFNβ reporter activity (relative to WT)
Chr9:32466433C>G	c.2192G>C	p.Gly731Ala	GGA	GCA	24.2	0.308	likely_benign	0	GOF
Chr9:32466433C>T	c.2192G>A	p.Gly731Glu	GGA	GAA	25.7	0.97	likely_pathogenic	6.24E-07	LOF
Chr9:32466434C>G	c.2191G>C	p.Gly731Arg	GGA	CGA	25.5	0.973	likely_pathogenic	Novel (this report)	LOF
Chr9:32466434C>T	c.2191G>A	p.Gly731Arg	GGA	AGA	25.7	0.973	likely_pathogenic	0	LOF
Chr9:32466433C>A	c.2192G>T	p.Gly731Val	GGA	GTA	25.3	0.836	likely_pathogenic	0	Not tested
	-	p.Gly731Leu	GGA	CTA	-	0.926	likely_pathogenic	-	GOF
	-	p.Gly731Phe	GGA	TTT	-	0.983	likely_pathogenic	-	Partial LOF
	-	p.Gly731Lys	GGA	AAA	-	0.991	likely_pathogenic	-	LOF

MAF: Minor Allele Frequency, CADD: Combined Annotation Dependent Depletion, LOF: Loss Of Function, GOF: Gain Of Function
