# Supplementary material for: Human RIG-I Antiviral Deficiency Caused by a Dominant-Negative Variant Locked in a Signaling-Inactive State
Source: medRxiv. 2026 Mar 6:2026.03.02.26347088. Preprint. [Version 1] doi: 10.64898/2026.03.02.26347088 (PMC12976907; doi:10.64898/2026.03.02.26347088)
Supplement: Supplement 2 [file media-2.pdf]

Potential Compound heterozygous model

gene	biotype	chrom	start	end	ref
GPR84	protein_codin	chr12	54757430	54757431	A
GPR84	protein_codin	chr12	54757552	54757554	AC
NBEAL1	protein_codin	chr2	203964368	203964369	G
NBEAL1	protein_codin	chr2	203972819	203972820	G
CDON	protein_codin	chr11	125887164	125887165	G
CDON	protein_codin	chr11	125831575	125831576	T
ARSD	protein_codin	chrX	2835988	2835989	A
ARSD	protein_codin	chrX	2835994	2835995	C
ARSD	protein_codin	chrX	2836040	2836041	A
ARSD	protein_codin	chrX	2836046	2836047	C
CTD-3088G3.8	protein_codin	chr16	11501174	11501175	C
CTD-3088G3.8	protein_codin	chr16	11572453	11572454	C

Autosomal dominant model

gene	biotype	chrom	start	end	ref
UBAP2	protein_codin	chr9	33944385	33944386	G
DDX58*	protein_codin	chr9	32466433	32466434	C
AIMP1	protein_codin	chr4	107252977	107252978	G
PANK2	protein_codin	chr20	3888722	3888723	C
TGM1	protein_codin	chr14	24729877	24729878	T
WDR5	protein_codin	chr9	137007134	137007135	C
CYP19A1	protein_codin	chr15	51507380	51507381	T
DRG2	protein_codin	chr17	17997260	17997261	C
PDS5B	protein_codin	chr13	33332328	33332329	A
CSMD2	protein_codin	chr1	34090704	34090705	G
BOK	protein_codin	chr2	242499105	242499106	C
C12orf66	protein_codin	chr12	64609581	64609582	C
MBD6	protein_codin	chr12	57919565	57919566	T
TECPR2	protein_codin	chr14	102891422	102891423	G
PIPOX	protein_codin	chr17	27381611	27381612	A
KIAA1239	protein_codin	chr4	37448444	37448445	C
UBALD2	protein_codin	chr17	74266472	74266473	T
KIAA1522	protein_codin	chr1	33237891	33237892	T
SEL1L3	protein_codin	chr4	25759185	25759186	A
TMEM194A	protein_codin	chr12	57453806	57453807	G
ABCB4	protein_codin	chr7	87053250	87053251	A
PKHD1L1	protein_codin	chr8	110499001	110499002	G
HEATR1	protein_codin	chr1	236715384	236715385	G
A2ML1	protein_codin	chr12	9027042	9027043	A
ZNF717	protein_codin	chr3	75786765	75786766	T

PHLPP1	protein_codin	chr18	60383070	60383071	A
SLC25A15	protein_codin	chr13	41367414	41367415	C
ANKRD6	protein_codin	chr6	90333715	90333716	T
OR52M1	protein_codin	chr11	4566991	4566992	C
FOXE3	protein_codin	chr1	47882525	47882526	C
ZNF292	protein_codin	chr6	87968685	87968686	C
WHSC1	protein_codin	chr4	1955152	1955153	A
DAPK1	protein_codin	chr9	90283552	90283553	C
ARHGEF38	protein_codin	chr4	106580386	106580387	C
R3HDML	protein_codin	chr20	42966024	42966025	T
ZNF736	protein_codin	chr7	63809516	63809517	A
TAF15	protein_codin	chr17	34171786	34171787	G
THNSL2	protein_codin	chr2	88472861	88472862	T
FIGNL1	protein_codin	chr7	50514362	50514363	C
CTD-3088G3.8	protein_codin	chr16	11572453	11572454	C
AFG3L2	protein_codin	chr18	12377022	12377023	T
THAP2	protein_codin	chr12	72058653	72058654	G
ZNF318	protein_codin	chr6	43306268	43306269	C
HUG1	protein_codin	chr10	102883591	102883592	A
PRMT2	protein_codin	chr21	48071771	48071772	C
CEACAM6	protein_codin	chr19	42265216	42265217	C
ZNF304	protein_codin	chr19	57867583	57867584	T
ECSIT	nonsense_me	chr19	11621446	11621447	C
NRDE2	protein_codin	chr14	90755266	90755267	A
CYP21A2	protein_codin	chr6	32007321	32007322	G
CUX2	protein_codin	chr12	111537439	111537442	GGT
CGNL1	protein_codin	chr15	57839672	57839673	C
TARSL2	protein_codin	chr15	102241343	102241344	C
PTBP1	protein_codin	chr19	798541	798542	G
TOM1L2	protein_codin	chr17	17765687	17765688	C
C21orf2	protein_codin	chr21	45750749	45750750	G
CCDC116	protein_codin	chr22	21989215	21989230	GCCTGGCTA(
CHADL	protein_codin	chr22	41634042	41634044	CG
FOXD4	protein_codin	chr9	117168	117170	AT
ICA1L	protein_codin	chr2	203653630	203653633	CAG
KRTAP4-9	protein_codin	chr17	39261807	39261857	GTCTGTGTGC
NLRP14	protein_codin	chr11	7078931	7078933	AT
PCSK5	protein_codin	chr9	78790206	78790207	C
RNF25	protein_codin	chr2	219528797	219528799	TC
ATN1	protein_codin	chr12	7045890	7045894	ACAG
C16orf3	protein_codin	chr16	90095597	90095598	G

CGNL1	protein_codin	chr15	57839674	57839675	C
HRCT1	protein_codin	chr9	35906594	35906595	A
KRT10	protein_codin	chr17	38975102	38975103	A
RP1L1	protein_codin	chr8	10467636	10467637	T

summarized WGS analysis for the patient based on those additional filters:

All genetic models: genotyping Quality score > 95

Recessive models: variants without no homozygous individuals in gnomAD database

Dominant models: variants are novel and not present in gnomAD database

No other IFN pathway variant found in the patient other than the RIG-I variant. No homozygous

GEMINI pipeline used for this analysis is using a different isoform (ENST00000379868.1) for RI

alt	impact	impact_severi	rs_ids	aaf_gnomad_	gnomad_num	gnomad_num
G	missense_var	MED	rs949956011	3.2329E-05	-1	-1
A	frameshift_va	HIGH	rs759103894	3.2369E-05	0	15
T	missense_var	MED	rs201657702	0.0002268	0	35
A	missense_var	MED	rs151000588	None	0	2
A	missense_var	MED	rs138087778	0.00029064	0	78
A	missense_var	MED	rs377706219	3.2285E-05	0	15
C	missense_var	MED	rs143238998	0.00035694	0	1
A	missense_var	MED	rs150899882	0.00035681	0	1
T	missense_var	MED	rs67272620	0.00042906	0	9
T	missense_var	MED	rs67359049	0.00042915	0	8
T	missense_var	MED	rs184717665	0.00353019	0	39
G	missense_var	MED	None	None	-1	-1

alt	impact	impact_severi	rs_ids	aaf_gnomad_	gnomad_num	gnomad_num
A	missense_var	MED	rs146320733	None	-1	-1
G	missense_var	MED	None	None	-1	-1
T	missense_var	MED	None	None	-1	-1
G	missense_var	MED	None	None	-1	-1
C	missense_var	MED	None	None	-1	-1
T	missense_var	MED	None	None	-1	-1
C	missense_var	MED	None	None	-1	-1
G	missense_var	MED	None	None	-1	-1
G	missense_var	MED	None	None	-1	-1
A	missense_var	MED	None	None	-1	-1
A	missense_var	MED	None	None	-1	-1
A	missense_var	MED	None	None	-1	-1
A	missense_var	MED	None	None	-1	-1
C	missense_var	MED	None	None	-1	-1
G	missense_var	MED	None	None	-1	-1
G	missense_var	MED	None	None	-1	-1
A	missense_var	MED	None	None	-1	-1
A	missense_var	MED	None	None	-1	-1
G	missense_var	MED	None	None	-1	-1
A	missense_var	MED	rs100658318	None	-1	-1
T	missense_var	MED	None	None	-1	-1
T	missense_var	MED	None	None	-1	-1
C	missense_var	MED	None	None	-1	-1
C	missense_var	MED	None	None	-1	-1
G	missense_var	MED	None	None	-1	-1

G	missense_var	MED	rs11152356	None	-1	-1
T	missense_var	MED	None	None	-1	-1
A	missense_var	MED	None	None	-1	-1
A	missense_var	MED	None	None	-1	-1
G	missense_var	MED	None	None	-1	-1
T	missense_var	MED	None	None	-1	-1
G	missense_var	MED	None	None	-1	-1
G	missense_var	MED	None	None	-1	-1
A	missense_var	MED	None	None	-1	-1
G	missense_var	MED	None	None	-1	-1
C	missense_var	MED	None	None	-1	-1
A	missense_var	MED	None	None	-1	-1
C	missense_var	MED	None	None	-1	-1
T	missense_var	MED	None	None	-1	-1
G	missense_var	MED	None	None	-1	-1
A	missense_var	MED	None	None	-1	-1
T	missense_var	MED	None	None	-1	-1
T	missense_var	MED	None	None	-1	-1
G	missense_var	MED	rs937838297	None	-1	-1
G	missense_var	MED	None	None	-1	-1
T	missense_var	MED	None	None	-1	-1
A	missense_var	MED	None	None	-1	-1
T	missense_var	MED	rs931413131	None	-1	-1
G	missense_var	MED	None	None	-1	-1
A	splice_accept	HIGH	None	None	-1	-1
G	splice_donor_	HIGH	None	None	-1	-1
CTGAGT	stop_gained	HIGH	None	None	-1	-1
T	missense_var	MED	rs143491081	None	0	0
A	missense_var	MED	rs568643597	None	0	0
T	missense_var	MED	rs962451906	None	0	0
A	missense_var	MED	rs749720437	None	-1	-1
G	frameshift_va	HIGH	None	None	-1	-1
C	frameshift_va	HIGH	None	None	-1	-1
A	frameshift_va	HIGH	None	None	-1	-1
C	frameshift_va	HIGH	None	None	-1	-1
G	frameshift_va	HIGH	None	None	-1	-1
A	frameshift_va	HIGH	None	None	-1	-1
CGAATA	frameshift_va	HIGH	rs71372053,r	None	-1	-1
T	frameshift_va	HIGH	None	None	-1	-1
A	inframe_delet	MED	None	None	-1	-1
GGGGCAGC(	inframe_inser	MED	None	None	-1	-1

CTGA	inframe_inser	MED	None	None	-1	-1
ACCACCCCC	inframe_inser	MED	rs762598108	None	-1	-1
AGCTGCCGC	inframe_inser	MED	None	None	-1	-1
TCCTCTAACT	inframe_inser	MED	rs369606728	None	-1	-1

s candidate for the patient.
IG-I so in the list, the variant is listed as p.Gly528Arg instead of p.Gly731Arg.

gnomad_num	is_coding	codon_chang	aa_change	aa_length	exon	vep_hgvsc
-1	1	Tac/Cac	Y/H	69/396	2/2	ENST0000026
250466	1	Gtg/tg	V/X	28/396	2/2	ENST0000026
156036	1	ttG/ttT	L/F	372/2694	11/55	ENST000004
157674	1	Gcc/Acc	A/T	591/2694	13/55	ENST000004
251288	1	cCg/cTg	P/L	249/1264	6/20	ENST0000026
249114	1	cAg/cTg	Q/L	1225/1287	19/20	ENST000003
123485	1	tTt/tGt	F/C	240/593	5/10	ENST000003
123661	1	tGc/tTc	C/F	238/593	5/10	ENST000003
118743	1	Ttc/Atc	F/I	223/593	5/10	ENST000003
117299	1	Ggt/Agt	G/S	221/593	5/10	ENST000003
6206	1	Gag/Aag	E/K	2236/2491	45/50	ENST000005
-1	1	Ggg/Cgg	G/R	731/2491	16/50	ENST000005

gnomad_num	is_coding	codon_chang	aa_change	aa_length	exon	vep_hgvsc
-1	1	Cgg/Tgg	R/W	508/1119	14/29	ENST0000036
-1	1	Gga/Cga	G/R	528/722	15/17	ENST000003
-1	1	Gtc/Ttc	V/F	181/312	5/7	ENST000003
-1	1	tCt/tGt	S/C	260/570	2/7	ENST000003
-1	1	Acg/Gcg	T/A	179/817	4/15	ENST000002
-1	1	aCc/aTc	T/I	110/334	5/14	ENST000003
-1	1	Atg/Gtg	M/V	303/503	8/10	ENST0000026
-1	1	Ctg/Gtg	L/V	67/364	2/13	ENST000002
-1	1	cAa/cGa	Q/R	1054/1447	27/35	ENST000003
-1	1	Ctc/Ttc	L/F	689/1167	13/24	ENST000003
-1	1	Ctg/Atg	L/M	70/212	2/5	ENST000003
-1	1	Gtt/Ttt	V/F	133/468	2/4	ENST000003
-1	1	cTg/cAg	L/Q	272/1003	6/13	ENST000003
-1	1	gGg/gCg	G/A	249/1411	6/20	ENST000003
-1	1	tAt/tGt	Y/C	237/390	5/8	ENST000003
-1	1	gCt/gGt	A/G	1612/1742	7/7	ENST000003
-1	1	Tcc/Acc	S/T	128/164	3/3	ENST000003
-1	1	Tcc/Acc	S/T	979/1035	6/7	ENST000003
-1	1	Tgg/Cgg	W/R	1042/1097	23/24	ENST0000026
-1	1	aCg/aTg	T/M	397/444	9/9	ENST000003
-1	1	Ttt/Att	F/I	728/1286	17/28	ENST0000026
-1	1	Gca/Tca	A/S	3278/4243	59/78	ENST000003
-1	1	aCt/aGt	T/S	2006/2063	43/44	ENST0000036
-1	1	tAc/tCc	Y/S	1415/1454	34/36	ENST000002
-1	1	Acg/Ccg	T/P	663/907	5/5	ENST000004

-1	1	gAg/gGg	E/G	52/1717	1/17	ENST0000026
-1	1	gCa/gTa	A/V	18/301	2/7	ENST0000033
-1	1	caT/caA	H/Q	386/727	12/16	ENST0000033
-1	1	aCa/aAa	T/K	191/317	1/1	ENST0000036
-1	1	cCa/cGa	P/R	180/319	1/1	ENST0000033
-1	1	gCt/gTt	A/V	1775/2718	8/8	ENST0000033
-1	1	tAc/tGc	Y/C	95/713	2/12	ENST0000038
-1	1	caC/caG	H/Q	655/1430	19/26	ENST0000035
-1	1	aaC/aaA	N/K	470/777	10/14	ENST0000042
-1	1	agT/agG	S/R	76/253	1/5	ENST0000021
-1	1	Aag/Cag	K/Q	426/427	5/5	ENST0000035
-1	1	gGt/gAt	G/D	492/589	15/16	ENST0000031
-1	1	Tct/Cct	S/P	65/484	1/8	ENST0000032
-1	1	gGt/gAt	G/D	208/674	4/4	ENST0000035
-1	1	Ggg/Cgg	G/R	731/2491	16/50	ENST0000059
-1	1	cAg/cTg	Q/L	20/797	1/17	ENST0000026
-1	1	gGc/gTc	G/V	26/52	2/2	ENST0000054
-1	1	Gta/Ata	V/I	1823/2279	10/10	ENST0000036
-1	1	Agc/Ggc	S/G	353/362	1/1	ENST0000059
-1	1	Ctt/Gtt	L/V	237/284	7/7	ENST0000033
-1	1	gCt/gTt	A/V	162/344	3/6	ENST0000019
-1	1	cTg/cAg	L/Q	116/659	3/3	ENST0000028
-1	0	cGt/cAt	R/H	48/71	2/6	ENST0000059
-1	1	Tct/Cct	S/P	818/1164	11/14	ENST0000035
-1	0					ENST0000041
-1	0					ENST0000039
-1	1	-/TGAGT	-/*	1298-1299/1:	19/19	ENST0000028
237622	1	tGt/tAt	C/Y	422/802	10/19	ENST0000033
10	1	gGc/gAc	G/D	4/56	1/4	ENST0000058
3522	1	tGc/tAc	C/Y	35/39	2/2	ENST0000046
-1	1	Cct/Tct	P/S	199/255	6/7	ENST0000032
-1	1	CCTGGCTACT	PGYCP/X	289-293/613	4/5	ENST0000029
-1	1	ggC/gg	G/X	344/762	3/6	ENST0000021
-1	1	gAt/gt	D/X	317/439	1/1	ENST0000038
-1	1	tCT/t	S/X	388/482	11/13	ENST0000035
-1	1	TCTGTGTGCT	SVCCQPTCSF	57-73/210	1/1	ENST0000039
-1	1	Ttt/tt	F/X	773/1093	7/12	ENST0000029
-1	1	cga/cGAATAg	R/RIX	688/690	14/14	ENST0000037
-1	1	Gag/ag	E/X	421/459	10/10	ENST0000029
-1	1	CAG/-	Q/-	488/1190	5/10	ENST0000035
-1	1	ccc/ccTGCAG	P/PAACPVGC	51/117	1/1	ENST0000040

-1	1	gcc/gcTGAc	A/AD	1299/1302	19/19	ENST0000028
-1	1	cac/caCCAC(	H/HHPHR	104/115	1/1	ENST0000035
-1	1	-/AGCTCCGG	-/SSGGGYGG	561-562/584	7/8	ENST0000026
-1	1	gaa/gGGACTA	E/GTKVIEGLQ	1324/2400	4/4	ENST0000038

vep_hgvsp	cadd_raw	cadd_scaled
ENSP0000026	3.67	25.7
ENSP0000026	None	None
ENSP0000039	3.07	23.8
ENSP0000039	3.69	25.9
ENSP0000026	2.83	23.3
ENSP0000037	1.66	16.59
ENSP0000037	1.52	15.9
ENSP0000037	0.1	4.3
ENSP0000037	0.08	3.89
ENSP0000037	1.12	14.02
ENSP0000047	1.33	15.04
ENSP0000047	0.63	10.5

vep_hgvsp	cadd_raw	cadd_scaled
ENSP0000035	4.27	32
ENSP0000036	4.14	29.5
ENSP0000035	4.02	28.1
ENSP0000031	3.86	26.8
ENSP0000020	3.84	26.7
ENSP0000035	3.83	26.6
ENSP0000026	3.51	25.1
ENSP0000022	3.43	24.8
ENSP0000031	3.34	24.6
ENSP0000036	3.29	24.4
ENSP0000031	3.28	24.3
ENSP0000031	3.1	23.9
ENSP0000034	3.13	23.9
ENSP0000035	3.08	23.8
ENSP0000031	3.05	23.7
ENSP0000030	2.95	23.5
ENSP0000033	2.83	23.3
ENSP0000036	2.75	23.1
ENSP0000026	2.73	23.1
ENSP0000030	2.77	23.1
ENSP0000026	2.6	22.8
ENSP0000036	2.58	22.7
ENSP0000035	2.46	22.5
ENSP0000029	2.37	22.2
ENSP0000038	2.15	20.8

ENSP000002(	2.14	20.7
ENSP00000034	1.96	18.8
ENSP00000034	1.93	18.52
ENSP0000003!	1.89	18.18
ENSP0000003:	1.58	16.17
ENSP00000034	1.54	15.97
ENSP0000003	1.21	14.48
ENSP0000003!	1.12	14.01
ENSP0000004	1.1	13.87
ENSP0000002	1.08	13.75
ENSP00000034	0.96	12.99
ENSP0000003(	0.89	12.53
ENSP0000003:	0.84	12.16
ENSP00000034	0.75	11.45
ENSP0000004	0.63	10.5
ENSP000002(	0.56	9.9
ENSP00000044	0.19	5.7
ENSP0000003!	0.11	4.45
ENSP0000004	-0.03	2.15
ENSP0000003:	-0.08	1.58
ENSP0000001!	-0.12	1.2
ENSP0000002{	-0.34	0.27
ENSP000004(	-0.37	0.23
ENSP00000034	-0.51	0.08
18967.2:c.550	3.37	24.6
)7643.3:c.55+	None	None
ENSP0000002{	None	None
ENSP0000003:	4.05	28.5
ENSP000004(	0.37	8.01
ENSP000004(	0.2	5.85
ENSP0000003	0.34	7.71
ENSP0000002!	None	None
ENSP0000002	None	None
ENSP0000003	None	None
ENSP0000003!	None	None
ENSP0000003	None	None
ENSP0000002!	None	None
'6767.3:c.*8_	None	None
ENSP0000002!	None	None
ENSP00000034	None	None
ENSP0000003{	None	None

ENSP0000028[illegible]	None	None
ENSP0000034[illegible]	None	None
ENSP0000026[illegible]	None	None
ENSP0000037[illegible]	None	None
